# TCF7L2 Polymorphism rs7903146 (C/T) and Gestational Diabetes Influence on Obstetric Outcome: A Romanian Case–Control Study

**DOI:** 10.3390/ijms25074039

**Published:** 2024-04-04

**Authors:** Gheorghe Cruciat, Andreea Roxana Florian, Mariam-Suzana Chaikh-Sulaiman, Adelina Staicu, Gabriela Valentina Caracostea, Lucia Maria Procopciuc, Florin Stamatian, Daniel Muresan

**Affiliations:** 1 Mother and Child Department, Obstetrics and Gynecology I, “Iuliu Haţieganu” University of Medicine and Pharmacy Cluj-Napoca, 400347 Cluj-Napoca, Romania; cruciat@yahoo.com (G.C.); florianandreea@ymail.com (A.R.F.); mariam.sulaiman@gmail.com (M.-S.C.-S.); staicuadelina@gmail.com (A.S.); caracostea1@yahoo.com (G.V.C.); muresandaniel01@yahoo.com (D.M.); 2 Department of Medical Biochemistry, “Iuliu Haţieganu” University of Medicine and Pharmacy Cluj-Napoca, 400347 Cluj-Napoca, Romania; 3 Imogen Clinical Research Centre, 400347 Cluj-Napoca, Romania; florin_stamatian@imogen.ro

**Keywords:** gestational diabetes mellitus, rs7903146 (C/T) polymorphism, TCF7L2 gene, fetal macrosomia

## Abstract

Gestational diabetes mellitus (GDM) is one of the most frequent predictors of obstetric outcome among Romanian pregnant women. Thus, we aimed to investigate the role of rs7903146 (C/T) TCF7L2 gene polymorphism in the presence of GDM and to evaluate the influence on maternal-fetal outcomes in a cohort of pregnant women from Northern Transylvania. Our prospective case–control study was performed in a tertiary maternity center on 61 patients diagnosed with GDM and 55 normal pregnant patients. The patients were genotyped for rs7903146 (C/T) polymorphism of the TCF7L2 gene using the PCR-RFLP method between 24 and 28 weeks of gestation. The minor T allele was associated with a high risk of developing GDM (OR 1.71 [95% CI 0.82–3.59]) if both heterozygote and homozygote types were considered. Also, a higher risk of developing GDM was observed in homozygous carriers (OR 3.26 [95% CI 1.10–9.68]). Women with the TT genotype were more likely to require insulin therapy during pregnancy than other genotypes with a 5.67-fold increased risk ([1.61–19.97], *p* = 0.015). TT homozygote type was significantly associated with fetal macrosomia for birth weights greater than the 95th percentile (*p* = 0.034). The homozygous TT genotype is associated with an increased risk of developing GDM. Also, rs7903146 (C/T) TCF7L2 variant is accompanied by a high probability of developing insulin-dependent gestational diabetes mellitus (ID-GDM). The presence of at least one minor T allele was associated with a higher risk of fetal macrosomia.

## 1. Introduction

Nowadays, due to the rising rates of obesity and metabolic disorders among young women, GDM comes up as one of the most frequent complications that arise during pregnancy [1].

Both hyperglycemia and GDM have been significantly associated with adverse perinatal outcomes. The severity of maternal glucose metabolism disorders influenced the overall frequency of adverse outcomes, such as preeclampsia or gestational hypertension, preterm birth and (NICU) admission, fetal macrosomia, primary cesarean delivery and clinically diagnosed neonatal hypoglycemia [2,3].

Considering that type 2 diabetes mellitus (T2DM) is a multigenic disease, several genetic polymorphisms with a role in glucose homeostasis were subsequently studied in GDM patients [4]. Identified as a risk factor in T2DM, TCF7L2 is one of the latest mapped genes whose expression disrupts pancreatic islet function lowering insulin secretion [5,6]. Glucagon-like peptide-1 (GLP-1) balances blood sugars by stimulating insulin secretion and lowering glucagon levels [7]. 

Located on chromosome 10q25.3, TCF7L2 modifies the effect of incretins on insulin secretion by lowering sensitivity in beta cells. It subsequently elevates blood sugars and raises the risk for T2DM [8], but this is not due to a reduced secretion of GLP-1. Out of various polymorphisms present in TCF7L2 that have been linked to T2DM, one particularly was found to have a strong association with GDM in Caucasian women. TCF7L2 rs7903146 (C/T) has been associated with an increased risk for insulin necessity in pregnant women with impairing postprandial glucose homeostasis [9,10]. 

This study aims to investigate the potential contribution of TCF7L2 (C/T) polymorphism to GDM susceptibility and to evaluate its influence on maternal-fetal outcomes in this cohort of Romanian pregnant women. 

## 2. Results

### 2.1. Obstetrical Outcomes

Data regarding demographic characteristics and obstetric parameters are depicted in Table 1. We found that the newborns from diabetic mothers had a higher birth weight than those from the control group, about 57.4% being over the 90th percentile compared to about 30.9% in the control group. The chi-square test showed a significant relationship between maternal diabetic status and newborns’ weight (*p* = 0.015). As expected, fetal macrosomia was more frequently encountered in the diabetic mothers’ group (*p* = 0.005).

There were significant differences between the two groups regarding maternal complications, such as hypertensive disorder (*p* = 0.002), the failure of labor induction (*p* = 0.014) and shoulder dystocia and lacerations (*p* = 0.002). No significant differences were found between the groups regarding obstetric complications, like premature rupture of membranes, preterm labor, polyhydramnios, and fetal dystocia (*p* > 0.05). The perinatal complications, both maternal and fetal, are presented in Table 2. 

### 2.2. Genetic Analysis

We used the Hardy–Weinberg principle to identify the potential genotyping errors in the control group studied. The test failed to reject the null hypothesis, suggesting that both the genotype and allele frequencies are in equilibrium.

Homozygous TT patients had a 3.26-fold increased risk of developing GDM with a tendency to statistical significance (*p* = 0.074). There were significant differences observed between the two studied groups regarding the allele frequencies (GDM vs. non-GDM, 43.3% vs. 29.1%). There was a 1.87-fold (*p* = 0.016) increased risk of developing GDM in carriers of the T allele (Table 3).

Our analysis of the patients’ BMI based on their genotype revealed that the average BMI exceeded the normal weight range in all three instances. Notably, patients with the TT genotype exhibited the highest average BMI (30.83 ± 5.53), surpassing those with the CC genotype (29.84 ± 4.29) and the CT genotype (30.67 ± 4.47). Nevertheless, statistical analysis failed to identify statistically significant differences between BMI means and patient genotypes (*p* > 0.05).

The analysis of maternal and fetal complications depending on the genotype showed no significant association (*p* > 0.05). However, women with a TT homozygote genotype had polyhydramnios and premature rupture of membranes significantly more often than expected compared to the other two genotypes studied with *p* = 0.0093 at an alpha level of 0.017 (Bonferroni adjusted level). 

Considering the necessity of insulin therapy in the GDM group, women with the TT genotype were more likely to develop insulin-dependent diabetes (45.5%) compared to other genotypes ((22.7%—CC genotype, 31.8%—CT genotype) OR 5.67 [CI 95% 1.61–19.97], *p* = 0.005) (Table 4).

We also analyzed the relationship between the maternal genotype and birth weight percentile. The presence of at least one T minor allele was associated with a high risk of fetal macrosomia (OR 1.13 [95% CI 0.54–2.36]). When only the homozygote TT genotype was considered, an even higher risk of macrosomia was found (OR 2.12 [95% CI 0.82–5.80]). Statistical significance was obtained when the homozygote type was correlated with birth weight greater than the 95th percentile (OR 3.12 [95% CI 1.16–8.38], *p* = 0.034). 

## 3. Discussion

GDM is a worldwide health problem due to its high prevalence in recent years because of the global increase in the prevalence of type 2 diabetes and obesity. It is well known that this condition leads to serious long-term consequences for both mother and fetus. 

Considering the risk of a patient with a history of GDM to develop T2DM during her lifetime, it was concluded that these two pathologies have similar pathophysiological mechanisms, share the same risk factors and apparently the same genetic susceptibility. However, the genetic terrain of GDM is not so well known yet.

This study investigates the correlation between the TCF7L2 rs7903146 (C/T) gene polymorphism and the development of GDM in Caucasian women. Our research demonstrates significant differences between the studied groups regarding the alleles of rs7903146 polymorphism, in a recessive and homozygote model. There is a significant correlation between the presence of the T allele and the development of GDM. Patients with at least one T allele, carriers of the heterozygous and homozygous genotypes, had 1.71 times higher risk of developing GDM. Patients with the TT genotype had 3.26 times higher risk compared with CC or CT genotypes. 

Rs 7903146 (C/T) variant of the TCF7L2 gene is known as the most frequently related polymorphism to GDM. The TCF7L2 gene is expressed in most tissues and organs, especially those involved in metabolic homeostasis, such as the liver, brain and subcutaneous adipose tissue. However, its role other than in the pancreas tissue remains uncertain [11]. A potential adverse effect of the presence of minor T allele of polymorphism rs7903146 (C/T) on insulin secretion has been suggested. However, the consequences for the pancreatic cellular function of this polymorphism remain to be clarified. It has been observed that the presence of this allele increases the expression of the TCF7L2 gene in the β-pancreatic cell with a decrease in insulin secretion [12]. 

Several studies have posited that TCF7L2, a significant gene implicated in the etiology of type 2 diabetes mellitus (T2DM), could potentially disrupt GLP1, diminish the expression of GLP1R and glucose-dependent insulinotropic polypeptide/gastrointestinal inhibitory peptide (GIP) receptor (GIP-R) and impede β-cell function through the stimulation of insulin secretion (in concert with GIP). TCF7L2 may also contribute to aberrant insulin processing and activation of the Wnt signaling pathway, culminating in the onset of T2DM [13,14].

Similar results have been obtained by a series of more recent studies showing that there are significant correlations between TCF7L2 SNP variants and the occurrence of GDM, as well as the existence of genetic variations related to ethnicity and race, making it necessary to validate this association for each population.

In contrast, Klein et al. (2012) found no significant correlation between the TT homozygous variant of the rs7903146 (C/T) TCF7L2 polymorphism and the occurrence of GDM [15,16,17,18,19,20]. 

The polymorphism investigated has been confirmed as a risk factor for GDM in both our study and in a recent study conducted in the Indian population, thus emphasizing the significance of TCF7L2 variants as potential biomarkers for evaluating disease risk irrespective of ethnicity and race [21]. The rs7903146 SNP variant, recognized as a risk factor among individuals in the American and Asian populations as well as in the Pacific region, might demonstrate a protective effect within the African population [22]. 

We also analyzed the presence of the risk allele to maternal-fetal complications and the need for insulin therapy during pregnancy. We demonstrated that the presence of the TT genotype is accompanied by a high probability of developing an ID-GDM compared to the CC and CT genotypes. 

Nevertheless, the analysis of maternal and fetal complications according to the genotype showed no significant association. However, women with the risk allele were more likely to develop insulin-dependent diabetes than other genotypes (*p* = 0.015).

Some studies have observed that under a hypocaloric diet, there is a lower expression of the TCF7L2 gene in adipose tissue in normoglycemic homozygous carriers of the T allele. On the other hand, Potasso et al. reported that the presence of the T allele of the rs7903146 (C/T) polymorphism is associated with inadequate insulin secretion, making insulin treatment necessary in patients with GDM in pregnancy [9]. Decreased insulin secretion in patients with the TT mutant genotype was also noted by Lyssenko et al [23]. 

A recent study points out that TCF7L2 rs7903146 polymorphism leads to altered glucose and lipid metabolism among patients with GDM. Also, those with risk allele T presented significantly higher 1 h blood glucose levels in OGTT compared to the non-risk genotypes. They concluded that insulin therapy must be taken into consideration when hyperglycemia cannot be properly controlled [24]. 

In recent years, research has revealed a correlation between the TCF7L2 rs7903146 polymorphism and compliance with a Mediterranean diet, impacting the onset of GDM through a gene–lifestyle interaction. Nutritional intervention alters the risk of GDM development exclusively among carriers of the T-risk allele and only in those women with moderate to high compliance, who experienced a lower GDM risk compared to those with low compliance [10]. 

In contrast, another study indicates a link between a different SNP (MTNR1B rs10830963) and a heightened propensity for insulin therapy among a cohort of patients diagnosed with GDM [25]. The research trends regarding the *MTNR1B* polymorphism have been shown to modify the outcome of a lifestyle intervention aiming to prevent GDM in pregnant women.

The research trends suggest that the MTNR1B polymorphism alters the efficacy of lifestyle interventions designed to prevent GDM in pregnant women. Although the genetic risk in women with GDM is known to intervene in the effectiveness of diet and lifestyle, the therapeutic approach is less well-established [26]. 

The findings of our study strengthen the evidence that the presence of the TT genotype is accompanied by a higher probability of developing ID-GDM than other genotypes (OR 5.67; 95% CI [1.61–19.97], *p* = 0.005). Furthermore, the presence of the minor T allele in both heterozygote and homozygote types was associated with an increased risk of fetal macrosomia.

Our results showed that the fetuses born from diabetic mothers had a higher birth weight than those from the control group (*p* = 0.015) and fetal macrosomia was more frequent in the diabetic mothers’ group (*p* = 0.005). Homozygote-type TT was associated with a higher risk of fetal macrosomia (*p* = 0.034). 

The results of our study agree with those published by Freathy et al., who concluded that each maternal copy of the T allele at rs7903146 increased offspring birth weight. They suggest that the most likely mechanism is reduced maternal insulin secretion, resulting in maternal hyperglycemia and increased insulin-mediated fetal growth [27]. 

The latest review underscores the importance of genetic testing and understanding the genetic profile of prospective mothers to anticipate potential unwanted complications. Considering that preterm birth, pregnancy-induced hypertensive disorders and GDM are on the rise, the future trend of prenatal care is shifting towards preventive medicine, placing emphasis on disease prevention rather than treating an already established condition. In this regard, genetics will occupy a central position in monitoring pregnancies and implementing personalized treatment strategies in the future [28]. 

Advancements in molecular technologies have spurred heightened endeavors to pinpoint the genes linked to susceptibility to GDM and to devise molecular-based approaches aimed at preventing and managing the condition [22].

To the best of our knowledge, this is the first study on this topic carried out on pregnant patients in Romania. 

The strength of our research is its applicability in daily practice, which improves the selection of patients at risk of developing GDM who would be responsive to dietary interventions according to their genetic characteristics and better identification of those at risk of developing a more severe form of this disease. 

However, there are several limitations to our study. Firstly, the size of the study groups cannot provide a significant result. Studies with larger sample sizes should be performed for a better understanding of the effects of rs7903146 (C/T) polymorphisms on GDM. 

Although our results hold significance, it is crucial to acknowledge that the sample size and regional specificity of the study could restrict the applicability of the findings. Subsequent research with broader and more varied populations is warranted to validate these conclusions.

Another potential weakness is the determination of only one polymorphism TCF7L2 rs7903146 in patients with GDM.

Future studies should investigate further genetic markers, such as the MTNR1B polymorphism, and their interplay with lifestyle factors to achieve a more comprehensive understanding of the risks associated with GDM.

Also, a limiting factor could be the missing data related to insulin regime therapy or maternal serum insulin levels in carriers of the at-risk allele. 

Further research on this topic, including bigger sample size, testing more polymorphisms of this gene, analyses between TCF7L2 rs7903146 and factors such as BMI, diet and physical activity are needed to conclude the genetic pattern of pregnant Romanian women.

## 4. Materials and Methods 

### 4.1. Study Design 

Between January 2018 and July 2020, we conducted a prospective case–control study, in Obstetrics-Gynecology Clinic I from Cluj-Napoca, a tertiary academic maternity center to which high-risk pregnancies and preterm neonates from central and western regions of Romania are referred.

The study was conducted in accordance with the Declaration of Helsinki and approved by the Bioethics Commission of the “Iuliu Haţieganu” University of Medicine and Pharmacy in Cluj-Napoca, Romania (No. 247/08.06.2017). The patients who participated in this research signed informed consent. We ensured that we had all their clinical data before starting the study. 

Our study included 116 consecutive women patients, aged 18–40 years, between 24 and 28 weeks of gestation, who agreed to the study protocol (OGTT testing, follow-up visits and delivery in our maternity).

Exclusion criteria were: T1DM (type 1 diabetes mellitus), T2DM (type 2 diabetes mellitus), acute or chronic infectious conditions, multiple pregnancies, stillbirth, chromosomal abnormalities or congenital anomalies and history of preterm delivery or preeclampsia.

The oral glucose tolerance test (OGTT) was performed with 75 mg glucose intake (after 8 h of fasting). GDM was diagnosed according to International Association of the Diabetes and Pregnancy Study Groups (IADPSG) criteria: when one of the three determined values was altered: fasting blood glucose > 92 mg/dL (5.1 mmol/L), 1 h glycemia > 180 mg/dL (10 mmol/L), 2 h glycemia > 153 mg/dL (8.5 mmol/L) [29]. 

According to the OGTT results, 61 patients were diagnosed with GDM. The other 55 patients were non-GDM, and we included them in the control group.

Two examinators assessed fetal well-being and intrauterine growth monthly through clinic and ultrasound examinations. The diabetologist managed women diagnosed with GDM according to the necessity of insulin therapy.

Structured questionnaires and hospital medical records provided the information regarding maternal age, body mass index (BMI) at study admission, weight gain during pregnancy, smoking habits, obstetrical and medical history during pregnancy and birth information (gestational age at delivery, route of delivery, labor induction, shoulder dystocia, soft tissue lacerations, premature rupture of membranes, preterm labor, birth weight, APGAR score at 5 min, perinatal morbidity and mortality). 

Macrosomia was considered the fetal growth beyond an absolute birth weight of 4000 g or above the 90th percentile. Intrauterine growth restriction (IUGR) was defined as low birth weight below the 10th percentile, regardless of gestational age [30]. 

### 4.2. Blood Collection and Biochemical Assays 

Maternal blood samples were collected between 24 and 28 weeks of gestation according to standardized procedures for performing OGTT. Venous blood samples were collected from peripheral vessels into commercially available EDTA Vacutainer tubes to determine the genotypes for rs7903146 (C/T) in the TCF7L2 gene. Blood samples were stored at −20 °C until analysis. 

### 4.3. rs7903146 (C/T) Polymorphism Genotyping

The identification of the C/T polymorphism located in intron 3 of the TCF7L2 gene (rs7903146) was performed by amplifying the genomic DNA using polymerase chain reaction (PCR) method, followed by the digestion of the amplified fragments with specific restriction endonucleases (Restriction Fragment Length Polymorphism (RFLP) technique).

For the isolation of genomic DNA from peripheral leukocytes, a ZymoResearch isolation kit (Quick-DNAMinipRep, Kit-Zymo Research Corporation, Freiburg, Germany) was used.

The PCR amplification of the fragment of interest, which contains the polymorphism rs7903146 (C/T), was achieved according to the method described by Kiliś-Pstrusińska et al. (2014) and optimized in the Biochemistry laboratory of UMF Cluj-Napoca in a total volume of 25 μL. It had the following amplification components: 20 ng genomic DNA, 200 μM deoxynucleoside triphosphates (dNTPs), 0.2 μM primers sense and antisense, 2.0 mM MgCl_2_, 2U *Taq polymerase* in buffer 50 mM KCl, 10 mM Tris-HCl [pH 8.4]. The primers used had the following sequences: primer sens 5′-GAG AGC TAA GCA CTT TTT AGG TA-3′ and primer antisense 5′-CTG ACA TTG ACT AAG TTA CTT GC-3′ (Jena Bioscience GmbH Loebstedter Strasse 80 D-07749, Jena, Germany) [31]. 

The amplification was performed in an iCycler (Bio-Rad Life Science, Hercules, CA, USA) according to the following program: initial denaturation at 94 °C for 5 min, followed by 34 amplification cycles that comprised denaturation at 94 °C for 0.6 s, hybridization of primers at 62 °C for 0.3 s and final elongation at 72 °C for 5 min. The amplified fragment has 113 bp. The amplified product obtained by PCR was digested with 5U *RsaI* restriction endonuclease (Jena Bioscience GmbH Loebstedter Strasse 80 D-07749, Jena, Germany) in 10 μL mixture for 3 h at 37 °C. rs7903146 (C/T) polymorphism destroyed a restriction site for the *RsaI* enzyme. The C allele gave after enzymatic digestion two fragments of 91 and 22 bp, while the T allele gave an undigested fragment of 113 bp.

**Figure 1:**
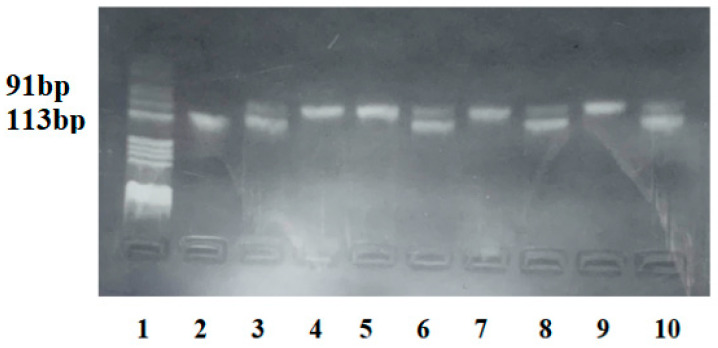
**Agarose gel electrophoresis for identification of rs7903146 polymorphism.** Lane 1—pBRHaeIII Digest DNA molecular marker; Lane 2—homozygous TT genotype: fragment of 113bp; Lanes 3, 6, 8, 10—heterozygous CT genotype: fragments of 113 and 91 bp; Lanes 4, 5, 7, 9—homozygous CC genotype: fragment of 91 pb.

To visualize the fragments obtained by amplification and enzymatic digestion, we performed electrophoresis in 3% agarose gel stained with 10 mg/mL ethidium bromide and visualization in UV light (Figure 1). 

### 4.4. Statistical Analysis

Statistical analysis was performed using IBM SPSS Statistics version 26.0 software and Microsoft Excel version 16.51. The continuous variables were characterized in terms of mean ± standard deviation. The qualitative data were described by frequency and percentage. The Pearson correlation coefficient was used to verify correlations between quantitative variables in the same group. The analysis of variance helped determine whether there were any differences between the means in the two groups. When needed, the association between different variables and the two groups studied was evaluated by the chi-square test, followed by post hoc tests. The odds ratios were also calculated to measure the association between the group variables. The Hardy–Weinberg principle was performed to determine whether the genotype and allele frequencies are in equilibrium within the population. The significance level was considered lower than 0.05, with new Bonferroni-adjusted levels when post hoc tests were conducted.

## 5. Conclusions

This study demonstrates that the presence of the TT genotype (rs7903146) represents an increased risk of developing GDM. The presence of this genotype is accompanied by a higher probability of developing insulin-dependent gestational diabetes compared to CC and CT genotypes. In this cohort of pregnant women, the presence of at least one minor T allele was associated with a higher risk of fetal macrosomia. 

## Figures and Tables

**Table 1:** The demographic data and obstetric parameters.

	GDM ^a^ (*n* = 61)	Non-GDM (*n* = 55)	*p*-Value ^d^
	(arithmetic mean ± SD ^b^)		
Maternal age (years)	31.9 ± 3.88	30.53 ± 5.07	0.102
BMI ^c^ at admission	31.49 ± 4.58	29.06 ± 4.24	**0.004**
Weight gain during pregnancy (kg)	15.05 ± 5.58	15.89 ± 5.24	0.418
Gestational age at birth (weeks)	38.44 ± 1.68	39.31 ± 1.17	**0.002**
Family history of diabetes no (%)	16 (26.2%)	8 (14.5%)	0.119
Smoking habit, no (%)	10 (16.4%)	2 (3.6%)	**0.021**
Parity, no (%)			0.737
Nulliparous	42 (68.9%)	39 (70.9%)	
Multiparous	19 (31.1%)	16 (29.1%)	
Delivery route no (%)			0.581
Vaginal	29 (47.5%)	29 (52.7%)	
C-section	32 (52.5%)	26 (47.3%)	
APGAR score at 5 min			1.000
≥7	59 (96.7%)	53 (96.4%)	
<7	2 (3.3%)	2 (3.6%)	
Birth weight (centiles)			**0.015**
≤p10	3 (4.9%)	3 (5.5%)	
p10–p90	23 (37.7%)	35 (63.6%)	
≤p90	35 (57.4%)	17 (30.9%)	

^a^ GDM—gestational diabetes mellitus; ^b^ SD—standard deviation; ^c^ BMI—body mass index; ^d^ significant *p*-values are noted in bold print.

**Table 2:** The maternal-fetal outcomes in the patient cohorts included in the study.

	GDM (N = 61)	Non-GDM (N = 55)	*p*-Value
**Maternal complications, (%)**			
Hypertensive pathology	12 (19.7%)	1 (1.8%)	**0.002**
Premature rupture of membranes	5 (8.2%)	0	0.059
Preterm birth	6 (9.8%)	1 (1.8%)	0.117
Polyhydramnios	5 (8.2%)	0	0.059
Failure of labor induction	7 (11.5%)	0	**0.014**
Shoulder dystocia/soft tissue lacerations	12 (19.7%)	1 (1.8%)	**0.002**
Fetal dystocia	4 (6.6%)	1 (1.8%)	0.367
**Fetal complications, (%)**			
IUGR	3 (4.9%)	3 (5.5%)	1.000
Macrosomia	26 (42.6%)	12 (21.8%)	**0.019**
Perinatal asphyxia/acute fetal distress	0	2 (3.6%)	0.223

**Table 3:** Distribution of rs7903146 (C/T) genotypes of TCF7L2 gene and allele frequencies in pregnant women with or without GDM.

TCF7L2 rs7903146 Genotype	Frequencies		*p*-Value ^b^	OR [95%CI] ^c^
	GDM ^a^ (N = 61)	Non-GDM (N = 55)		
CC, no (%)	23/61 (37.7%)	28/55 (50.9%)	0.074	0.58 [0.28–1.22]
CT, no (%)	23/61 (37.7%)	22/55 (40%)		0.91 [0.43–1.92]
TT, no (%)	15/61 (24.6%)	5/55 (9.1%)		3.26 [1.10–9.68]
Alleles				
C allele, no (%)	69/122 (56.6%)	78/110 (70.9%)	**0.016**	0.53 [0.31–0.92]
T allele, no (%)	53/122 (43.3%)	32/110 (29.1%)		1.87 [1.09–3.23]
Dominant model				
CC, no (%)	23/61 (37.7%)	28/55 (50.9%)	0.107	0.58 [0.28–1.22]
CT+TT, no (%)	38/61 (62.3%)	27/55 (49.1%)		1.71 [0.82–3.59]
Recessive model				
TT, no (%)	15/61 (24.6%)	5/55 (9.1%)	**0.024**	3.26 [1.10–9.68]
CC+CT, no (%)	46/61 (75.4%)	50/55 (90.1%)		0.31 [0.10–0.91]
Co-dominant model				
CT, no (%)	23/61 (37.7%)	22/55 (40%)	0.475	0.91 [0.43–1.92]
CC+TT, no (%)	38/61 (62.3%)	33/55 (60%)		1.1 [0.52–2.33]
Homozygote				
CC, no (%)	23/38 (60.5%)	28/33 (84.8%)	**0.021**	0.27 [0.09–0.87]
TT, no (%)	15/38 (39.5%)	5/33 (15.2%)		3.65 [1.15–11.57]
Heterozygote				
CC, no (%)	23/48 (50%)	28/50 (56%)	0.351	0.79 [0.35–1.76]
CT, no (%)	23/48 (40%)	22/50 (44%)		1.27 [0.57–2.84]

^a^ GDM—gestational diabetes mellitus; ^b^ significant *p*-values are noted in bold print; ^c^ OR—odds ratio; CI—confidence interval.

**Table 4:** The statistical analysis of the genotype frequencies of the rs7903146 (C/T) polymorphism in the TCF7L2 gene among diabetic patients with and without insulin requirement.

GDM Type	Insulin-Requiring GDM	Non-Insulin-Requiring GDM	OR (95% CI)
CC	5 (22.7%)	18 (46.2%)	0.34 (0.11–1.12)
CT	7 (31.8%)	16 (41%)	0.67 (0.22–2.02)
TT	10 (45.5%)	5 (12.8%)	5.67 (1.61–19.97)
*p*-value	**0.015**		

## Data Availability

The datasets analyzed during the current study are not publicly available being part of the ECCN Cluj-Napoca, Romania archives but are available from the corresponding author on reasonable request.
